# Multilingual hope speech detection from tweets using transfer learning models

**DOI:** 10.1038/s41598-025-88687-w

**Published:** 2025-03-15

**Authors:** Muhammad Ahmad, Iqra Ameer, Wareesa Sharif, Sardar Usman, Muhammad Muzamil, Ameer Hamza, Muhammad Jalal, Ildar Batyrshin, Grigori Sidorov

**Affiliations:** 1 Centro de Investigación en Computación, Instituto Politécnico Nacional (CIC-PN), 07738 Mexico City, Mexico; 2 Department of Computer Science, Division of Engineering and Science at Abington, The Pennsylvania State University, University Park, PA 19001 USA; 3 Department of Computer science, Artificial Intelligence, and Software Engineering, The Islamia University of Bahawalpur, Bahawalpur, 63100 Pakistan; 4 School of Informatics and Robotics, Institute of Arts and Culture, Lahore, 54000 Pakistan

**Keywords:** Hope speech, Deep learning, Transfer learning, Bert, Roberta, SVM, Social media, Machine learning, And Twitter analysis, Computational science, Mathematics and computing, Information technology

## Abstract

Social media has become a powerful tool for public discourse, shaping opinions and the emotional landscape of communities. The extensive use of social media has led to a massive influx of online content. This content includes instances where negativity is amplified through hateful speech but also a significant number of posts that provide support and encouragement, commonly known as hope speech. In recent years, researchers have focused on the automatic detection of hope speech in languages such as Russian, English, Hindi, Spanish, and Bengali. However, to the best of our knowledge, detection of hope speech in Urdu and English, particularly using translation-based techniques, remains unexplored. To contribute to this area we have created a multilingual dataset in English and Urdu and applied a translation-based approach to handle multilingual challenges and utilized several state-of-the-art machine learning, deep learning, and transfer learning based methods to benchmark our dataset. Our observations indicate that a rigorous process for annotator selection, along with detailed annotation guidelines, significantly improved the quality of the dataset. Through extensive experimentation, our proposed methodology, based on the Bert transformer model, achieved benchmark performance, surpassing traditional machine learning models with accuracies of 87% for English and 79% for Urdu. These results show improvements of 8.75% in English and 1.87% in Urdu over baseline models (SVM 80% English and 78% in Urdu).

## Introduction

Recently, people have been spending more time on social media platforms, making decisions more frequently based on sentiments shared by these online communities. Social media platforms allow us to monitor the activities of our friends and family in a manner similar to how we would in our everyday lives. In addition, it enables us to connect with peoples we have never met in person from around the world. On these platforms, the predominant sentiments fall into two categories: hope and hate. Hope is a positive state of mind characterized by the anticipation of favorable outcomes in one’s life events and circumstances. Hope can be valuable for individuals aiming to sustain a steady and positive perspective on life^1^. We often use hopeful phrases, such as “Great job!“, “Kudos to you!” and “Continue the excellent work” to motivate and encourage others. In contrast, hate expresses a negative sentiment prevalent on online platforms that seeks to harass individuals based on characteristics such as race, religion, ethnicity, sexual orientation, disability, or gender^2^. The primary objective of social media platforms is to reduce the dissemination of hateful content, while actively encouraging and amplifying positive and hopeful expressions. Hope speech plays a crucial role in promoting positivity, fostering resilience, and encouraging individuals to maintain an optimistic outlook, which can lead to healthier, more constructive interactions. By highlighting and spreading hope, we can help combat negativity, creating a more supportive and uplifting online environment.

Hope speech detection is a relatively new approach that aims to identify and promote positive content, foster harmony, and encourage a positive atmosphere in society. Among the limited studies on hope speech detection, most have focused on monolingual contexts, where the model is trained and tested within a single language, such as English^3^, Tamil and Malayalam^4^, Kannada^5^, Bengali^6^, and Spanish^7^. The LT-EDI-EACL 2021^1^ shared task made a significant contribution to Hope Speech Detection by organizing competitions in English, Tamil, and Malayalam, advancing research on identifying positive and supportive content across these languages. However, no significant research has addressed hope speech detection in Urdu, either within monolingual or multilingual frameworks. To address this, we adopted a “multilingual” with translations based approach for hope speech detection. The concept of “multilingual hope speech detection (MHSD)” involves a comprehensive approach to processing mixed-language texts commonly found in social media conversations within multilingual communities, such as English and Urdu in Pakistan or similar countries, where both languages are widely used in public discourse. Translation-based techniques are employed to bridge linguistic gaps and accurately detect hope speech in this mixed-language content. This approach enables effective sentiment analysis and fosters positive discourse in multilingual online communities.

Recent developments in natural language processing (NLP) have provided new techniques to advance hope speech detection task. Models like BERT and its multilingual variants, as discussed by Devlin et al.^8^ and Pires et al.^9^, have demonstrated impressive performances in capturing semantic and contextual information across several languages. These models leverage transfer learning to fine-tune language representations pre-trained on specific tasks, enabling sophisticated identification of hope speech^10^.

This study highlights the potential of machine learning, deep learning, and advanced language models in promoting positive online discourse through the detection of hope speech. By focusing on both English and Urdu, it bridges the linguistic gap, ensuring that underrepresented communities are included in computational research. Urdu, spoken by over 230 million people globally^11^, plays a crucial role in social media interactions but has often been overlooked in natural language processing. In response, we developed a multilingual dataset for both English and Urdu, contributing to the emerging field of hope speech detection in these languages. Using a translation-based approach via the Google Translate API, we fine-tuned cutting-edge models to navigate the complexities of multilingual data. Our proposed models achieved (BERT) 87% accuracy on the English dataset and 79% on the Urdu dataset, demonstrating their capacity to identify and foster positive, constructive dialogues. By detecting and amplifying hope speech, this research not only counters the pervasive negativity on social media but also contributes to the creation of safer, more inclusive online spaces, where diverse voices are elevated and respected. This work underscores the broader societal value of cultivating optimism and civility in digital spaces, encouraging a more compassionate and constructive online culture.

The contributions of this paper are as follows:

1. Our findings indicate that multilingual and joint translation-based approaches have not previously been explored for English and Urdu datasets;
2. We explore the psychological basis of hope speech and conceptualize hope as a form of expectation, which informs the design of our dataset and the approach to classification tasks;
3. We propose, implement, and evaluate transfer learning tools that help users actively encourage and amplify positive and hopeful expressions;
4. Conduct a comprehensive analysis and performance evaluation by employing various learning techniques along with visualization methods;
5. The comprehensive set of experiments demonstrated that the proposed framework achieved benchmark-level performance, surpassing the baselines in joint-translated approaches.

The rest of the paper is organized into six sections: “Literature survey” reviews related work on Hope Speech detection. “Methodology and design” presents the methodology and design. Results and analysis are presented in “Results and analysis”. “Limitation of the proposed solution” present the limitations of the proposed solution. Finally, “Conclusion and future work” discusses the conclusions of our research and potential avenues for future exploration.

## Literature survey

Hope is essential for the well-being and recovery of individuals, as recognized by health professionals. It is defined as a positive mental state focused on anticipating favorable results in life’s situations or the world at large. This hopeful perspective is both future-oriented and driven by a desire for positive outcomes^12^.

Nath et al.^6^ highlights the lack of research on hope speech detection in Bengali, despite its significance due to the large number of Bengali speakers. They address this gap by creating a high-quality, annotated dataset of Bengali tweets and applying computational models to validate its effectiveness for hope speech research.

Daniel, et al.^7^. Focuses on hope speech, which can inspire positivity and ease hostile environments, as an alternative to detecting negative content. It introduces SpanishHopeEDI, a new Spanish Twitter dataset on the LGBT community, and provides baseline experiments for further research on hope speech detection.

Chakravarthi et al.^13^ introduces a multilingual dataset designed to recognize and promote positive comments, using a custom deep network architecture with T5-Sentence embedding. The proposed CNN model outperformed other machine learning models, achieving macro F1-scores of 0.75 for English, 0.62 for Tamil, and 0.67 for Malayalam.

Chakravarthi et al.^14^ introduce HopeEDI, a multilingual dataset of hope speech from YouTube, annotated in English, Tamil, and Malayalam. It emphasizes the need for positive online content and provides benchmarks using precision, recall, and F1-score. The dataset, available for public use, aims to foster further research in promoting inclusive and supportive speech.

Balouchzahi et al.^15^ introduce a hope speech English dataset that classifies tweets into “Hope” and “Not Hope,” with further categorization into three hope types such as realistic hope, unrealistic hope and generalized hope. It details the annotation process and evaluates various baseline, deep learning and transfer learning approaches, finding that contextual embedding models outperform simpler classifiers in detecting hope speech.

Daniel et al.^16^ focuses on developing new datasets and systems for the automatic detection of hope speech, primarily in Spanish, using classical machine learning and deep learning techniques. It emphasizes the importance of studying hope speech, which can inspire positivity, as an alternative to solely detecting hate speech.

Shahid Iqbal et al.^17^ explores multilingual hope speech detection in English and Russian using transfer learning and RoBERTA. It introduces a new Russian corpus and evaluates joint multi-lingual and translation-based approaches in binary classification. The translation-based method (Russian-RoBERTA) achieved the highest performance with 94% accuracy and 80.24% F1-score. Our work is different in Urdu and English language.

## Methodology and design

### Construction of dataset

**Fig. 1:**
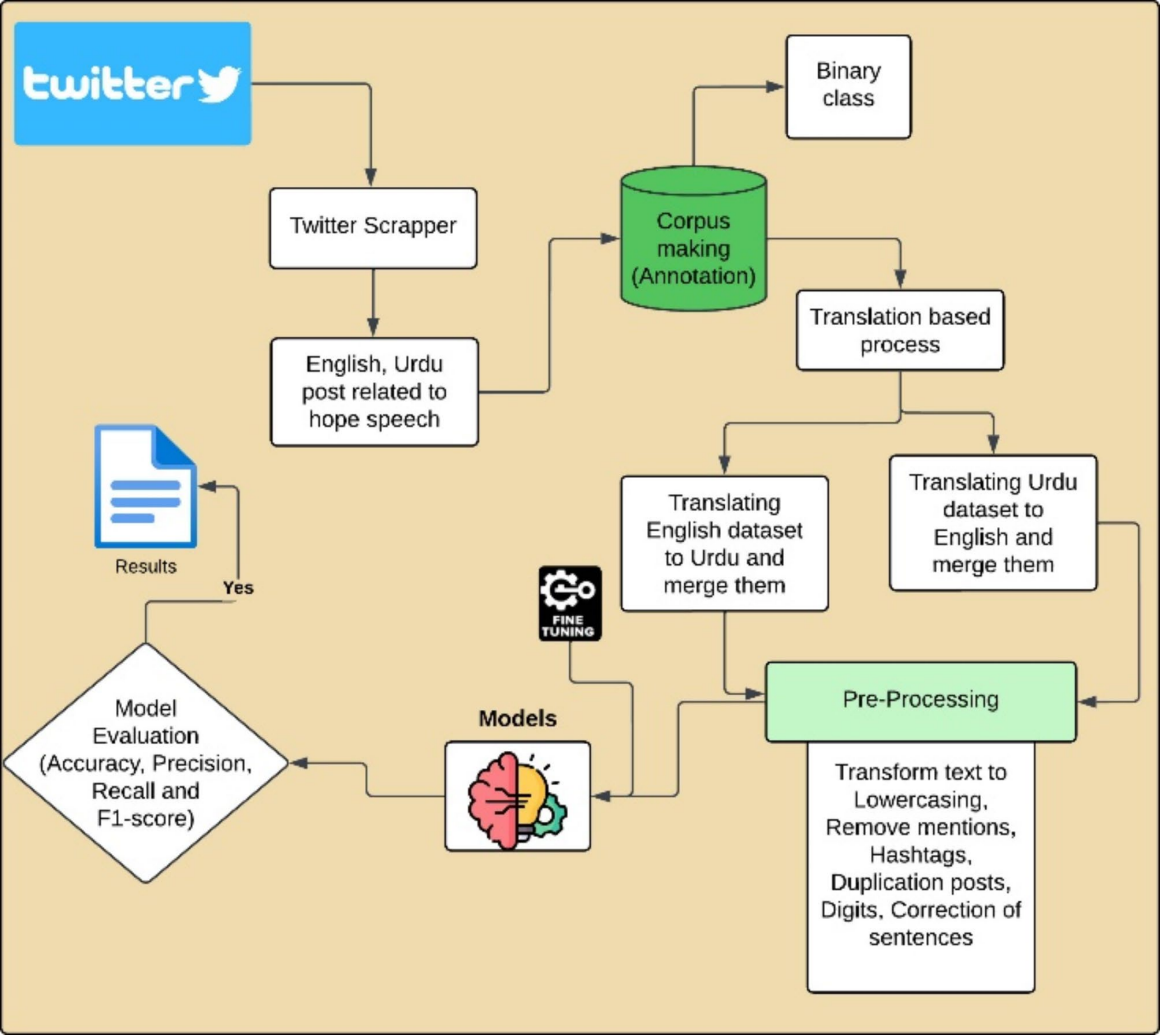
Proposed methodology and design.

This section outlines the data collection process for the hope speech detection using the Tweepy API, which is used to extract tweets from Twitter. The dataset consists of 25,000 Urdu and 18,000 English Tweets collected between January 2023 and March 2024 related to hope speech. To extract relevant tweets, we used specific keywords that were carefully selected for their potential to evoke strong emotions related to either hope or not hope. To capture expressions of hope, positive words such as for Urduامید (Umeed), حوصلہ (Housla) عزم (Azm) - خوشی (Khushi) مثبت (Masbat) etc. while in English such as “Congratulations,“, “Thank you,” “Brave,” “Hope,” and “Support” were used. Additionally, words associated with negative emotions, like “Lose,” “Ignorance,” “End,” “Sorrow,” and “War,” were included to gather non-hopeful. We incorporated different parts of speech (verbs, nouns, adjectives, and adverbs) using the same keywords to capture a broader range of relevant tweets. In total, 43,000 tweets were collected and saved in a 2 different CSV files for the further process. Figure 1 shows the proposed methodology and design of the study.

### Annotation

Annotation involves assigning labels to each sample through a manual evaluation process. In this study three independent annotators were selected to label each sample into binary classification such as a tweet as either “hope” or “not-hope.” Annotation is a crucial step for creating a high-quality dataset, so we carefully selected postgraduate students in Computer Science who were native speakers of both Urdu and English to ensure linguistic accuracy and cultural relevance in the annotation process. The final label for each tweet was determined based on majority voting.

#### Annotation guidelines

Annotation guidelines consist of a set of instructions provided to annotators to assist them in effectively classifying tweets as “hope” or “non-hope”. These guidelines are not strict rules, but serve to help annotators distinguish between hope and not hope speech. Although all annotators receive the same set of guidelines, their individual interpretations ultimately determine whether the qualities described are present in a tweet binary class, as shown in Table 1. The following criteria were established to identify tweets that should be marked as binary class hope-speech detection.

Hope

- Positive and encouraging tone that conveys optimism.
- Expresses belief in a better future, progress, or solutions.
- May offer support, motivation, or reassurance to keep going.
- The tweet includes supportive, hopeful, and empathetic responses during times of distress, tension, or uncertainty.
- It contains words of reassurance, encouragement, inspiration, compliments, or satisfaction in optimistic social contexts.
- It expresses tolerance towards hate.
- It offers supportive comments directed towards any minority community.
- It demonstrates faith in the possibility of a better future.
- The tweet includes expressions of appreciation for someone.
- It conveys gratitude or appreciation for other people, places, or circumstances.

Not hope

- Negative, neutral, or indifferent tone that lacks optimism.
- Focuses on challenges, obstacles, or failure without expressing belief in improvement.

**Table 1:** Examples of hope and not hope tweets.

Language	Tweets	Label
English	Even in the darkest times, there’s always a glimmer of hope. Keep believing, keep pushing, and never give up. Better days are coming!	Hope
	It feels like things aren’t improving, and I’m not sure where this is all headed	Not hope
Urdu	زندگی میں مشکلات آئیں گی، مگر ہمیشہ امید رکھیں کہ حالات بہتر ہوں گے۔	Hope
	English Translation Challenges will come in life, but always keep hope that things will get better	
	یہ حالات کبھی نہیں بدلیں گے، کوشش کرنے کا کوئی فائدہ نہیں۔	Not hope
	English Translation These circumstances will never change, there is no point in trying	

#### Annotation procedure

The labeling of tweets in the binary class followed the annotation guidelines outlined above. These guidelines, along with the tweet list, were provided to the three selected annotators. The list was given in a randomized order, and each annotator was tasked with labeling all (*n* = 9236) tweets in both Urdu and English as either “Hope” or “Not-hope,” based on the provided criteria. According to the classification guidelines, any tweet not evoking hope was labeled as “*Not-hope*,” including tweets that potentially carried neutral or negative sentiments. The annotators were given ample time to complete the task. To supervise the annotation process, individual Google Forms were created for each annotator to ensure consistency and track key annotation details. These forms were designed to capture categorization criteria, challenges faced, and any ambiguities encountered. Weekly meetings were scheduled to review the data collected through the forms, assess the progress of the annotation, and address any challenges encountered. Afterward, their annotations were reviewed and adjusted to ensure that the correct labels were assigned. Notably, in the binary classification, annotators 1 and 2 exhibited nearly identical labeling patterns. The distribution of the labels remained balanced throughout the corpus.

### Inter annotator agreement

We calculated the inter-annotator agreement (IAA) using Fleiss’ Kappa and pairwise Kappa for binary classifications. Fleiss’ Kappa^18^ is particularly useful when dealing with three or more annotators and categorical output labels. The dataset used in this study contained 9236 tweets, each labeled by three annotators. During the annotation process, differences in opinions among the annotators may arise, and it is essential to examine and analyze these discrepancies to derive meaningful insights from annotators’ outputs. This evaluation was conducted by calculating the IAA, which serves as a measure of the quality and reliability of the annotation process, indicative of the accuracy of the results^19^. Landis and Koch^20^ proposed a way to interpret agreement levels based on the value of K, which is given in Table 2, while the pairwise Kappa values between the annotators used in this study is shown in Table 3, reflect the level of agreement in their annotations. The Kappa value between Annotator 1 and Annotator 2 is 0.94, indicating strong agreement. The Kappa value between Annotator 1 and Annotator 3 is 0.78, suggesting moderate to strong agreement. Finally, the Kappa value between Annotator 2 and Annotator 3 is 0.74, indicating moderate agreement. These results demonstrate a high degree of consistency in the annotation process across all annotator pairs.

**Table 2:** Interpretation of the Kappa values.

Kappa value range	Interpretation
1.0	Perfect agreement
0.80 to 1.0	Substantial agreement
0.60 to 0.80	Moderate agreement
0.40 to 0.60	Fair agreement
< 40	Poor agreement

**Table 3:** Pairwise Kappa values between annotators.

Annotator pair	Kappa value
Annotator 1 and Annotator 2	0.94
Annotator 1 and Annotator 3	0.78
Annotator 2 and Annotator 3	0.74

### Corpus characteristics and standardization

**Fig. 2:**
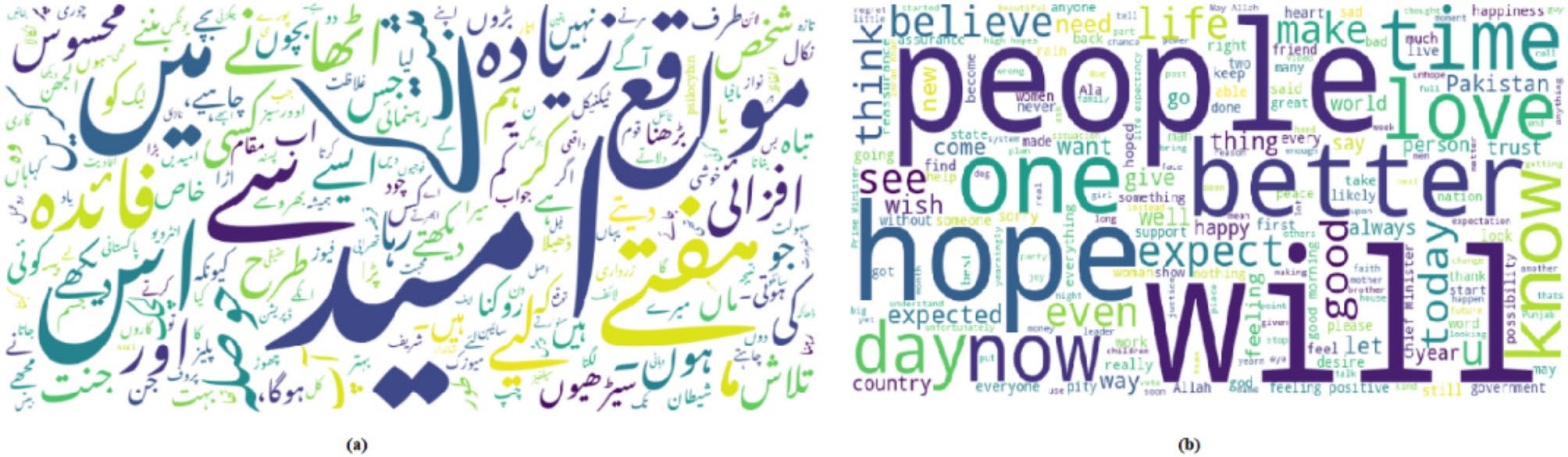
Word cloud (**a**) Urdu (**b**) English.

**Fig. 3:**
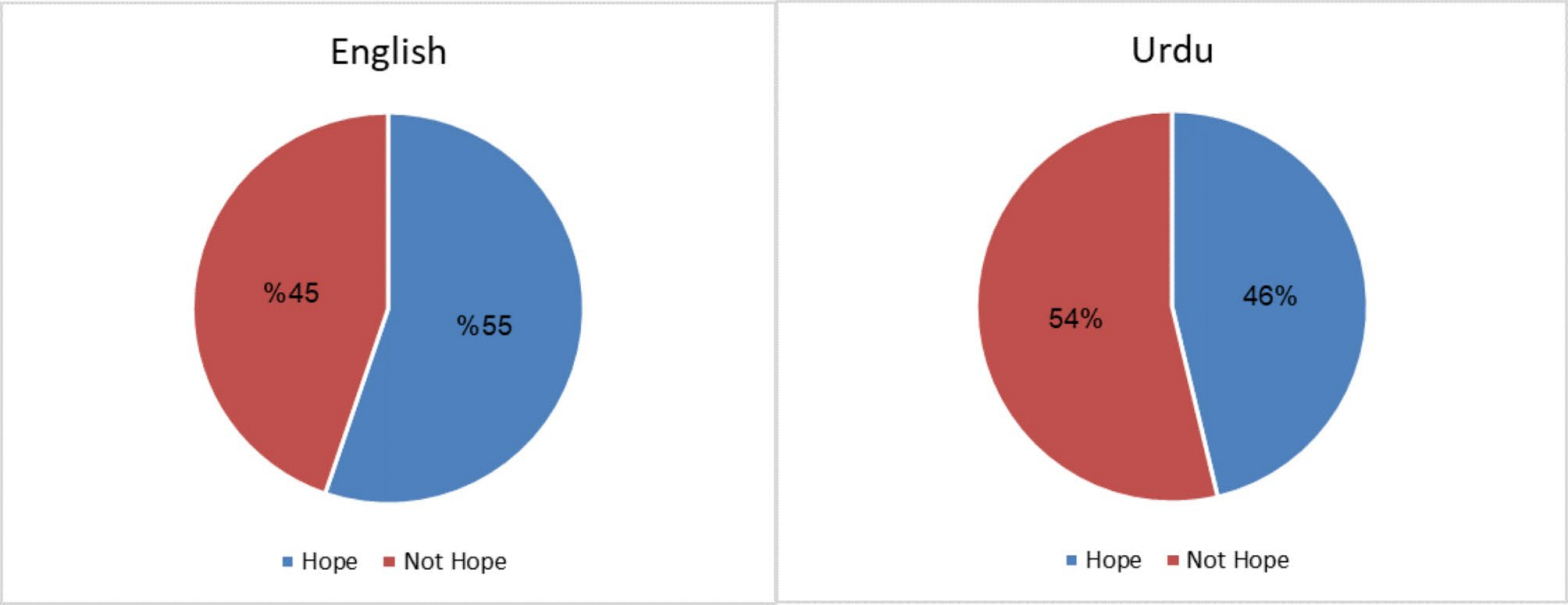
Label distribution (**a**) English Dataset and (**b**) Urdu Dataset.

**Fig. 4:**
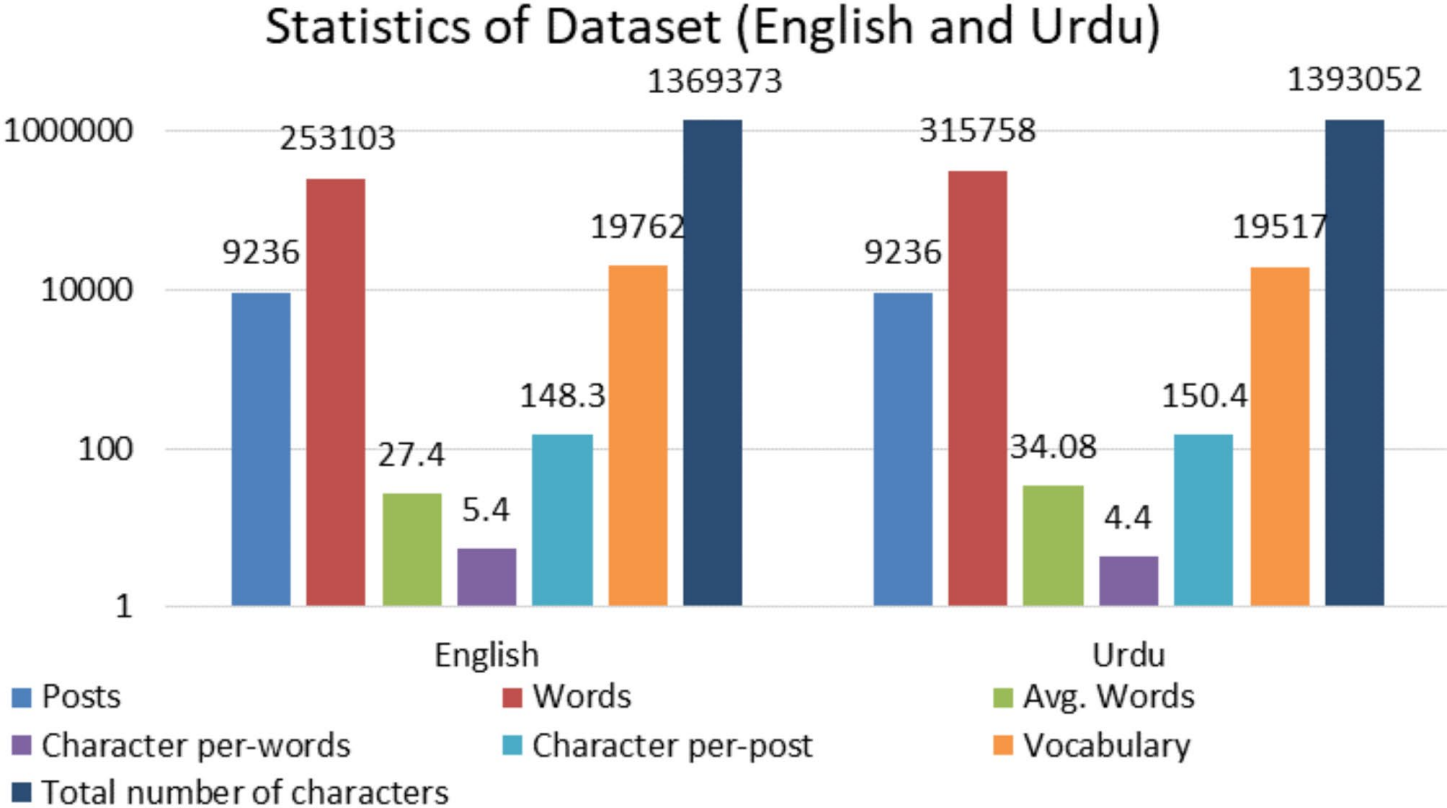
Dataset statistics in both Urdu and English languages.

The proposed corpus comprises 4934 English and 4302 Urdu samples, resulting in a total of 9,236 after translating between the two languages out of 4700 labeled as Hope and the remaining 4536 samples labeled as Not Hope. This balancing data is crucial for ensuring the best performance of the model during training and testing phase. Figure 2 illustrate the word clouds while Fig. 3 shows the data distribution for each language. Figure 4 illustrates key statistics of a dataset comprising both English and Urdu languages. It presents a comparison between the two languages across several metrics, including the number of posts, total words, and average words per post, vocabulary size, character count, and character-related averages. Both languages feature the same number of posts (9236), but English shows a higher total character count (1,369,373) compared to Urdu (1,393,052). Urdu, however, has a slightly larger vocabulary size (19,517 words) than English (19,762 words). The average number of words per post is higher for Urdu (34.08) compared to English (27.4), while English has a higher average number of characters per word (5.4) relative to Urdu (4.4). These statistics reflect the linguistic differences and variations in text structure between the two languages in the dataset.

### Ethical concern

Social media data, particularly concerning Racial and Ethnic Minorities, Religious Minorities, People with Disabilities and Economic Minorities, are highly sensitive. To minimize the risk of exposing individual identities, we carefully removed personal information such as names and religion from the dataset, with the exception of public figures, and pledged not to contact the original authors. The dataset will be made available only to researchers who agreed to comply with ethical guidelines for research.

### Translation based approach

The translation-based approach aims to standardize both Urdu and English texts by converting all content into a single language and storing the dataset in a single CSV file; in the first column of the CSV, there are tweets the 2nd column contain a label, making it easier to process and analyze. The translation pipeline involves the following steps:

- Pre-translation Tokenization: Before proceeding with the translation, the text is divided into smaller tokens called words or phrases. This step helps to improve translation accuracy by allowing translation tools to better understand the context and meaning of each segment.
- Handling Noisy Translations: Once translation is complete, we carefully manually review the entire procedure to track any errors, especially for idiomatic expressions or slang that may not translate smoothly between languages. This ensures that the translated content maintains its intended meaning and clarity.
- Post-Translation Alignment: To maintain consistency, we first translated the Urdu language text into English and combined it into a single CSV file to create a single corpus for 2nd corpus we translated the English language to Urdu texts carefully aligned with the original Urdu texts in the dataset. This ensures that both translated and native original texts are of similar quality for analysis.
- Text Length Standardization: For texts that are excessively long, truncation is applied to ensure a uniform input length, making the data more suitable for deep learning models.

This approach helps ensure that linguistic differences between Urdu and English do not interfere with model performance and enables uniform text processing across both languages. Table 4 represents the pseudo-code for multilingual hope speech classification (Urdu-English Dataset using BERT model).

**Table 4:** Multilingual hope speech detection classification (English dataset, Urdu dataset).

Step	Procedure/operation	Description
1	Multilingual. Hope-speech-classification ()	Procedure for multilingual hope speech classification (Urdu-English Dataset)
2	D1 ← Urdu-Dataset (4,302)	Load Urdu dataset (4302 samples)
3	D2 ← English-Dataset (4,934)	Load English dataset (4934 samples)
4	Eng-Translated-D1 ← Translate-To-English (D1)	Translate Urdu dataset into English
5	Urd-Translated-D2 ← Translate-To-Urdu (D2)	Translate English dataset into Urdu
6	Review-Translations (Eng-Translated-D1, Urd-Translated-D2)	Manually review translations for accuracy, especially focusing on idiomatic expressions or slang
7	Combined-Eng-D ← Merge (Eng-Translated-D1, D2)	Combine reviewed translated Urdu (in English) with the original English dataset
8	Combined-Urd-D ← Merge (Urd-Translated-D2, D1)	Combine reviewed translated English (in Urdu) with the original Urdu dataset
9	Augmented-Eng-D ← Back-Translation (Combined-Eng-D)	Apply back-translation to augment the combined English dataset (e.g., English → French → English)
10	Augmented-Urd-D ← Back-Translation (Combined-Urd-D)	Apply back-translation to augment the combined Urdu dataset (e.g., Urdu → Arabic → Urdu)
11	Pre-Processed-Eng-D ← Pre-Processing (Augmented-Eng-D)	Pre-process the augmented English dataset (Cleaning, Lower-Case, Replace Emoji)
12	Pre-Processed-Urd-D ← Pre-Processing (Augmented-Urd-D)	Pre-process the augmented Urdu dataset (Cleaning, Lower-Case, Replace Emoji)
13	Com-D1 ← BERT-Tokenizer (Pre-Processed-Eng-D, Pre-Processed-Urd-D, ‘bert-base-multilingual-cased’)	Tokenize the combined and augmented dataset (Urdu and English) using BERT multilingual model
14	Classification (Com-D1, 3)	Classify the combined and augmented dataset (Urdu-English) using a fine-tuned Multilingual BERT model
15	Pre-Processing(D)	Procedure to pre-process dataset (Cleaning, Lower-Case, Replace Emoji)
16	D1 ← Cleaning(D)	Remove hashtags, mentions, punctuation, URLs, numbers, etc.
17	D2 ← Lower-Case(D1)	Convert text to lower-case
18	D3 ← Replace-Emoji(D2)	Replace emojis/emoticons with corresponding text
19	Classification (Dataset D, mode)	Procedure for classification using fine-tuning of the relevant multilingual BERT model
20	Model ← fine-tuning (D, ‘Bert-base-multilingual-cased’, 80 − 20)	Fine-tune the combined and augmented Urdu-English dataset using BERT multilingual (cased) model (80 − 20 split)
21	confusion-matrix ← generate-results (Model)	Generate confusion matrix from the fine-tuned model
22	accuracy ← compute-accuracy(confusion-matrix)	Compute accuracy from the confusion matrix
23	precision ← compute-precision(confusion-matrix)	Compute precision from the confusion matrix
24	recall ← compute-recall(confusion-matrix)	Compute recall from the confusion matrix
25	F1-score ← compute-F1(confusion-matrix)	Compute F1-score from the confusion matrix

### Preprocessing

**Fig. 5:**
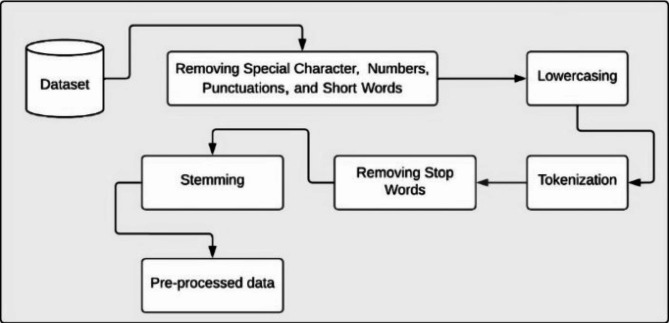
Data preprocessing approach.

Data pre-processing involves transforming raw data to make it suitable for machine learning models. It is essential for analyzing Twitter posts, to improve the quality of data and performance of the model. The preprocessing involves a series of essential steps, first, we standardize the input text by removing special character, numbers, punctuations, short word and extra spaces, converting everything to lowercase for uniformity. Next, we standardize the text into individual words or tokens, making it easier to work with. In the following step, we focus on removing noise from the tokens by looping through each one and filtering out any non-alphanumeric characters, ensuring that only relevant content remains. We then eliminate unwanted words by loading a list of English stop words^21^ and filtering out these common words, along with any tokens that are shorter than three characters. This helps refine our dataset further. Afterward, we apply stemming to the filtered tokens, which reduces words to their base forms, ensuring consistency across the dataset. To handle class imbalance, we applied SMOTE (Synthetic Minority Over-sampling Technique) to balance the classes in the dataset. SMOTE is a popular technique used to address class imbalance by generating synthetic samples for the minority class. By applying SMOTE, we ensure a more reliable evaluation of the model, as it mitigates the bias caused by imbalanced data. Finally, we compile the cleaned and processed tokens into a final list, ready for analysis as seen Fig. 5. Through these carefully executed steps, we ensure that the text data is clean, relevant, and well-prepared for meaningful insights.

### Feature extraction

After effectively translating and pre-processing our dataset, we turned our attention to feature extraction. This step is crucial because it enables the conversion of textual data into numerical formats in which machine learning algorithms can work effectively. For machine learning, we used the Term Frequency–Inverse Document Frequency (TF-IDF) as the feature extraction method. For deep learning, we used FasText and GloVe, and for transfer learning, we used transformer-based language models leveraging contextual embeddings as the feature extraction method.

#### TF-IDF

TF-IDF is a technique used to determine how often a word appears in a document, while also reducing the influence of words that appear in multiple documents across the corpus. The advantage of using TF-IDF lies in its simplicity of calculation and ability to assess the relevance of keywords effectively.

1 $$\:TF=\frac{\text{N}\text{u}\text{m}\text{b}\text{e}\text{r}\:\text{o}\text{f}\:\text{t}\text{i}\text{m}\text{e}\text{s}\:\text{t}\text{e}\text{r}\text{m}\:\text{t}\:\text{a}\text{p}\text{p}\text{e}\text{a}\text{r}\text{s}\:\text{i}\text{n}\:\text{a}\:\text{d}\text{o}\text{c}\text{u}\text{m}\text{e}\text{n}\text{t}}{\text{T}\text{o}\text{t}\text{a}\text{l}\:\text{n}\text{u}\text{m}\text{b}\text{e}\text{r}\:\text{o}\text{f}\:\text{t}\text{e}\text{r}\text{m}\text{s}\:\text{i}\text{n}\:\text{t}\text{h}\text{e}\:\text{d}\text{o}\text{c}\text{u}\text{m}\text{e}\text{n}\text{t}}$$

The IDF of a term reflects the inverse proportion of documents containing that term. Terms with technical jargon, for example, hold greater significance than words found in only a small percentage of all documents. The IDF can be computed using the following Eq. (2);

2 $$\:IDF=\frac{\text{N}\text{u}\text{m}\text{b}\text{e}\text{r}\:\text{d}\text{o}\text{c}\text{u}\text{m}\text{e}\text{n}\text{t}\:\text{i}\text{n}\:\text{t}\text{h}\text{e}\:\text{c}\text{o}\text{r}\text{p}\text{u}\text{s}}{\text{N}\text{u}\text{m}\text{b}\text{e}\text{r}\:\:\text{o}\text{f}\:\text{d}\text{o}\text{c}\text{u}\text{m}\text{e}\text{n}\text{t}\:\text{i}\text{n}\:\text{t}\text{h}\text{e}\:\text{c}\text{o}\text{r}\text{p}\text{u}\text{s}\:\text{c}\text{o}\text{n}\text{t}\text{a}\text{i}\text{n}\:\text{t}\text{e}\text{r}\text{m}\text{s}}\:\:$$

TF-IDF can be calculated in Eq. (3);

3 $$\:TF-IDF\:=TF\times\:IDF\:$$

#### FasText

FastText extends Word2Vec by representing words as bags of character n-grams. The embedding for a word ‘w’ is calculated in Eq. (4);

4 $$\:Vw=\sum\:_{g\in\:G\left(w\right)}Vg$$

where G (w) is the set of character n-grams in the word w. Vg is the vector representation of each n-gram g.

#### GloVe

GloVe (Global Vectors for Word Representation) creates word embedding’s based on the co-occurrence matrix of words. The key Eq. (5) is derived from the ratio of co-occurrence probabilities:

5 $$\:Cost=\sum\:_{i,j}^{V}f\left({X}_{i,j}\right){{{(V}_{i}}^{T}{V}_{j}+{b}_{i}+{b}_{j}-{log}{(x}_{i,j}\left)\right)}^{2}$$

where X_i, j_​ is the number of times word j occurs in the context of word i. V is the vocabulary size. V_i_ and V_j_ are the embedding’s for words i and j. b_i_ and bj​ are bias terms for the words. f(X_i, j_) is a weighting function to down-weight the influence of very frequent words.

#### Transformer-based contextual embeddings

In addition to traditional and deep learning methods, we employed transformer-based language models for feature extraction in our transfer learning approach. These models generate contextualized embeddings, providing dynamic word representations based on their surrounding context. Unlike static embeddings like FastText and GloVe, contextual embeddings adapt to the specific context in which words appear, enabling a deeper semantic understanding of the text. TF-IDF, FastText, and GloVe were used alongside transformers to provide a comparative analysis because they each offer different strengths: TF-IDF highlights important terms based on frequency, while FastText and GloVe offer pre-trained word-level embeddings that capture semantic relationships. These traditional methods were contrasted with transformers’ contextual embeddings to understand their individual contributions to hope speech detection. Each of these feature extraction techniques—TF-IDF, FastText, GloVe, and transformer-based language models—was used separately to evaluate their individual impact on hope speech detection. This approach allowed us to analyze the effectiveness of statistical, deep learning, and contextual embedding methods independently for multilingual social media discourse.

### Application of models, training and testing phase

**Fig. 6:**
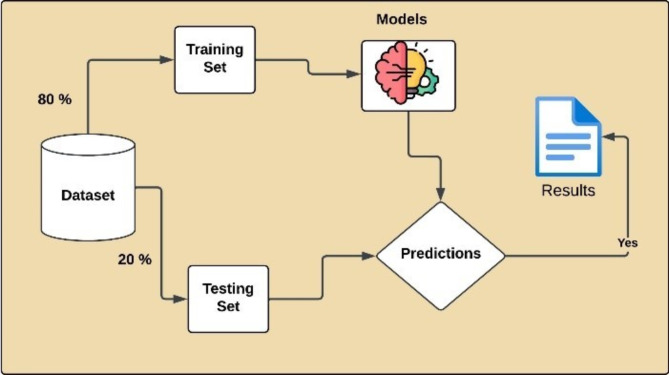
Application of models training and testing phase.

To optimize the models for the best performance, grid and random search techniques were used to explore different combinations of hyper parameters as shown in Table 5. A grid search systematically tests all possible combinations, whereas a random search selects random sets of hyper parameters. These tuning methods ensure that each model is configured to perform the classification tasks as accurately as possible. Specifically, we utilized two hope speech datasets: one in Urdu and another in English. To evaluate our datasets we applied state-of-the-art six machine learning models such as Extreme gradient boosting (XGB), Naive Bayes (NB), Logistic Regression (LR), Decision tree (DT), Random forest (RF) and Support vector machine (SVM) (using TF-IDF word embedding’s) and two deep learning models such as Convolutional Neural Network (CNN) and Bidirectional Long Short-Term Memory (BiLSTM) with two different word embedding’s (GloVe and FastText) and four transformer-based models such as Bidirectional Encoder Representations from Transformers (BERT) and Robustly Optimized BERT Approach (RoBERTa), Google Electra and GPT-2 for representation learning. The transformer-based models use pre-trained embedding’s to leverage rich contextual information and multilingual capabilities, enhancing the accuracy and robustness of the classification tasks across languages. For all experiments, we applied an 80–20 split, allocating 80% of the data for training and remaining 20% for testing. This split ensures that the models learn patterns from the training data while being evaluated on unseen data during testing to monitor the performance, as shown in Fig. 6.

The Table 5 presents a comparative overview of the learning approaches, models, and hyperparameter configurations used in this study. To address concerns regarding the misuse or lack of distinction between methodologies, we elaborate on the specific setups and parameters that differentiate each approach:

Transformer-Based Models: The transformer-based models (e.g., BERT, mBERT, RoBERTa, GPT-2, and ELECTRA) were fine-tuned using domain-specific data, a core application of transfer learning. These models were pre-trained on large corpora, and their learned knowledge was transferred to our task by fine-tuning them with carefully selected hyperparameters. For instance, a learning rate of 2e-5, 3 epochs, a batch size of 32, the AdamW optimizer, and CrossEntropyLoss function were chosen through grid search. This approach is aligned with transfer learning principles, which leverage the power of pre-trained models to reduce the need for large amounts of task-specific data while ensuring the models are adapted for specific tasks such as sentiment analysis and hope speech detection.

Fine-Tuning of mBERT and BERT: Both mBERT (Multilingual BERT) and BERT (Bidirectional Encoder Representations from Transformers) were fine-tuned in the context of our multi-language task. For mBERT, which is pre-trained on a large multilingual corpus, we used a similar fine-tuning process as BERT but adapted it for multilingual text. During fine-tuning, mBERT was exposed to our specific multilingual dataset containing text in Urdu, and English. The model’s parameters, such as the learning rate (2e-5), the number of epochs (3), and batch size (32), were selected to optimize performance for the specific classification task. This fine-tuning process ensures that the model effectively learns the nuances of each language, while leveraging pre-existing language knowledge from mBERT’s training.

For BERT, which was pre-trained on English text data, fine-tuning followed a similar process, but it was done exclusively with the English subset of our dataset. This adaptation ensures that the model is not only leveraging its pre-trained knowledge but also refining it to recognize patterns within our task-specific data. Fine-tuning was performed using task-specific labels (e.g., hope speech detection), and hyperparameters were selected to maximize model convergence and performance for the binary classification problem.

Traditional Machine Learning Models (Benchmarking Role): To provide a benchmark and distinguish them from the transformer-based deep learning models, we utilized traditional machine learning models like Support Vector Machines (SVM), XGBoost (XGB), Naïve Bayes (NB), DT, RF, and Logistic Regression (LR). It is important to note that these models are not transfer learning approaches, as they do not leverage pre-trained representations or fine-tuning of large-scale models. Instead, their role is to serve as a benchmark for comparison against the more advanced transformer-based models. By training them directly on the task-specific data without utilizing pre-trained models, we establish a baseline performance to evaluate the advantages and performance improvements offered by transfer learning. Each model’s hyperparameters, such as kernels for SVM, tree estimators for XGB, and solver choices for LR, were optimized using grid search to ensure rigorous comparison. This distinction clarifies how traditional machine learning methods differ from transfer learning models, where knowledge is adapted from pre-trained models.

Deep Learning Models: We used BiLSTM and CNN models to capture sequential and spatial patterns in text data. These models require extensive training from scratch and differ fundamentally from transfer learning approaches, as they do not benefit from pre-trained representations. Parameters such as embedding dimensions (300), learning rates (0.1), and batch sizes (32) were tuned using grid search. The use of these deep learning models emphasizes the contrast in methodological application compared to transformer-based models, where the core strength of transfer learning is its ability to fine-tune pre-trained knowledge to specific tasks.

By explicitly defining the hyperparameters and optimization processes for each model, this table underscores the distinction between transfer learning, traditional machine learning, and deep learning methodologies. This clarification ensures that the application of transfer learning is well justified and not misused, emphasizing its role in adapting pre-trained models for task-specific performance, while also providing a clear comparison with other methodologies.

**Table 5:** Optimum values identified for the hyper-parameters of proposed models.

Learning approach	Models	Hyper parameter	Grid search
Transformer	BERT, mBERT, RoBERTa, GPT-2, ELECTRA	Learning rate, epoch, batch size, Optimizer, Loss Function	2e-5, 3, 32, AdamW, CrossEntropyLoss
Machine learning	SVM	Random state, kernel, c value, gamma	42, linear and rbf, 1.0, auto
	XGB	n_estimators, max_depth, learning_rate	100, 6, 0.3
	DT	random state, max_depth, min_samples_split, min_samples_leaf	42, 10, 2, 1
	RF	n_estimators, max_depth, min_samples_split, min_samples_leaf	100, 10, 2, 1
	NB	Alpha, fit_prior, class_prior	1.0, true, none
	LR	Random state, max_iter, c value, solver	42, 1000, 0.1, liblinear
Deep learning	BiLSTM and CNN	Learning rate, epoch, embedding_dim, batch size,	0.1, 5, 300, 32

### Evaluation metrics

Accuracy: This metric calculates the percentage of correctly classified instances out of the total number of predictions, providing a straightforward measure of the model’s overall performance, as calculated in Eq. (6).

Precision: Precision evaluates the number of predicted positive cases (hope speech) that are correct. This is particularly important when the cost of false positives (incorrectly identifying non-hope speech as hope speech) is high, as calculated in Eq. (7).

Recall: Known as the sensitivity or true positive rate, recall measures how many of the actual positive cases (hope speech) are correctly identified by the model. This helps in understanding how well the model captures hope speech, as calculated in Eq. (8).

F1-score: F1-score is the harmonic mean of the precision and recall, offering a balanced measure when both precision and recall are important. This is especially useful when there is an imbalance in the dataset, ensuring that neither metric is disproportionately favored, as calculated in Eq. (9).

6 $$\:\text{A}\text{c}\text{c}\text{u}\text{r}\text{a}\text{c}\text{y}=\frac{\text{T}\text{N}+\text{T}\text{P}}{\text{T}\text{o}\text{t}\text{a}\text{l}\:\text{P}\text{r}\text{e}\text{d}\text{i}\text{c}\text{t}\text{i}\text{o}\text{n}\text{s}}$$

7 $$\:\text{P}\text{e}\text{r}\text{c}\text{i}\text{s}\text{i}\text{o}\text{n}=\frac{\text{T}\text{P}}{\text{F}\text{P}+\text{T}\text{P}}$$

8 $$\:\text{R}\text{e}\text{c}\text{a}\text{l}\text{l}=\frac{\text{T}\text{P}}{\text{F}\text{N}+\text{T}\text{P}}$$

9 $$\:\text{F}1-\text{s}\text{c}\text{o}\text{r}\text{e}=2\times\:\frac{\:\text{R}\text{e}\text{c}\text{a}\text{l}\text{l}\:\times\:\text{P}\text{r}\text{e}\text{c}\text{i}\text{s}\text{i}\text{o}\text{n}}{\text{R}\text{e}\text{c}\text{a}\text{l}\text{l}+\:\text{P}\text{r}\text{e}\text{c}\text{i}\text{s}\text{i}\text{o}\text{n}\:}$$

While TP is true positive, FP is false positive and TN is true negative.

## Results and analysis

### Experimental setup

Experiments were conducted on a Lenovo laptop powered by an Intel Core i7 8th generation processor with 4 cores, bus speed of 8 GT/s, 24 GB of RAM, and 1 TB of storage. The operating system used was Windows 10 Pro, which provided a stable environment for development and execution. To perform the predictive analysis Google Colab was selected for programming and easy access to a Python environment, we utilized Python version 3.12.4 and Scikit-Learn library, version 1.3.0, was employed to implement the machine learning algorithms. Moreover, using Google Colab’s GPU capabilities significantly enhances the performance of transformer-based models.

### Results for machine learning

Table 6 presents the performance of several traditional machine learning models using TF-IDF word embedding’s on both English and Urdu datasets, evaluated by Accuracy, Precision, Recall, and F1-score.

For the English dataset SVM stands out as the best performer, achieving the highest scores of 0.8 across Precision, Recall, F1-score, and Accuracy, indicating it is the most reliable model for this task. Both XGB and LR follow closely, with 0.79 across all metrics, making them strong alternatives to SVM, though slightly less effective. NB and RF score similarly, with 0.77 in most metrics, showing they are decent models but not as strong as the top three. DT, on the other hand, has the lowest performance with 0.72 for Accuracy and lower values for other metrics, suggesting that it is less suited for this classification problem. In conclusion, SVM emerges as the top model, while DT shows the weakest performance in comparison to the other models.

For the Urdu dataset LR and SVM both perform exceptionally well, achieving identical scores of 0.78 across Precision, Recall, F1-score, and Accuracy, making them the most reliable models for this task. XGB, NB, and RF all have similar performance, with 0.77 in all metrics, demonstrating they are effective but slightly less optimal than LR and SVM. DT, however, performs the weakest, with scores of 0.69 in Accuracy and lower values in the other metrics, indicating it struggles to handle the classification task effectively. In summary, LR and SVM stand out as the top models for Urdu, while DT remains the least effective option.

In summary, SVM outperformed all other models in both languages, while DT demonstrated consistent underperformance across both datasets. Other models like XGB, LR, and RF displayed competitive results.

**Table 6:** Results for machine learning.

Language	Models	Precision	Recall	F1-score	Accuracy
English	XGB	0.79	0.79	0.79	0.79
	LR	0.79	0.79	0.79	0.79
	NB	0.77	0.77	0.77	0.77
	RF	0.77	0.77	0.76	0.77
	DT	0.73	0.72	0.72	0.72
	SVM	0.8	0.8	0.8	0.8
Urdu	XGB	0.77	0.77	0.77	0.77
	LR	0.78	0.78	0.78	0.78
	NB	0.77	0.77	0.77	0.77
	RF	0.77	0.77	0.77	0.77
	DT	0.69	0.69	0.68	0.69
	SVM	0.78	0.78	0.78	0.78

### Deep learning results

Two deep learning models used in the experiments such as CNN and BiLSTM were trained with specific parameters. Each model was trained for 20 epochs per fold, using the Adam optimizer with a learning rate of 0.001. The loss function applied was categorical cross entropy, and both models utilized a dropout rate of 0.1 to prevent over-fitting. In the case of CNN, filters of sizes [1, 2, 3 and 5] were employed, with a total of 36 filters. Both models used an embedding size of 300 for feature representation and a soft-max activation function for classification.

Table 7 compares the performance of CNN and BiLSTM models using two different word embeddings—FasText and GloVe—on classification tasks for both English and Urdu languages, with metrics including Precision, Recall, F1-score, and Accuracy. For English, the BiLSTM model using FasText embedding performs the best, achieving scores of 0.81 across Precision, Recall, F1-score, and Accuracy, making it the most effective model. The CNN model with FasText follows closely with a score of 0.76, indicating good performance but not quite as strong as BiLSTM. When using GloVe embeddings, BiLSTM shows slightly lower performance (0.79 for F1-score and Accuracy), while CNN achieves 0.77, both still strong but lower than the FasText-based BiLSTM. For Urdu, the BiLSTM with FasText performs the best at 0.75 for Precision, Recall, and F1-score, with an Accuracy of 0.75, showing it is the most reliable model for this language as well. In contrast, CNN with FasText performs poorly with a Precision of 0.71, but the Recall, F1-score, and Accuracy are significantly lower, especially with GloVe embeddings. The CNN and BiLSTM models using GloVe in Urdu show even poorer results, with the highest Accuracy being 0.51 for CNN and 0.5 for BiLSTM, demonstrating that GloVe does not perform well for Urdu in this task. In conclusion, BiLSTM with FasText performs the best for both English and Urdu, while CNN models, particularly with GloVe, show significantly weaker results, especially for Urdu.

**Table 7:** Results for deep learning.

Language	Embedding’s	Models	Precision	Recall	F1-score	Accuracy
English	FasText	CNN	0.76	0.76	0.76	0.76
		BiLSTM	0.81	0.81	0.81	0.81
	GloVe	CNN	0.77	0.77	0.77	0.77
		BiLSTM	0.8	0.79	0.79	0.79
Urdu	FasText	CNN	0.71	0.51	0.35	0.51
		BiLSTM	0.75	0.75	0.74	0.75
	GloVe	CNN	0.54	0.51	0.37	0.51
		BiLSTM	0.5	0.5	0.34	0.5

### Transformers results

Table 8 compares the performance of four language models for text classification in English and Urdu. For English, bert-base-uncased leads the pack with the highest scores across all metrics at 0.87, showcasing its strength in understanding and classifying English text accurately. Close behind is roberta-base, achieving a solid 0.86 across the board, followed by gpt2, which performs reliably with scores of 0.85. Electra-base-discriminator has slightly lower performance, with an F1-score of 0.83, but it still demonstrates competence in handling English text.

For Urdu, the performance is more varied. Bert-base-multilingual-cased performs best, with an F1-score of 0.78 and accuracy of 0.79, making it the top choice for handling Urdu text. Gpt2 also shows strong performance, scoring 0.77 across most metrics. However, roberta-base underperforms compared to its English counterpart, achieving a consistent but lower score of 0.72. Electra-base-discriminator, in contrast, struggles significantly in Urdu, with an F1-score of just 0.33 and an imbalanced precision-recall performance, highlighting its difficulty in managing this language.

**Table 8:** Results for transformers models.

Language	Models	Precision	Recall	F1-score	Accuracy
English	**Bert-base-uncased**	**0.87**	**0.87**	**0.87**	**0.87**
	Roberta-base	0.86	0.86	0.86	0.86
	Electra-base-discriminator	0.84	0.84	0.83	0.84
	gpt2	0.85	0.85	0.85	0.85
Urdu	**Bert-base-multilingual-cased**	**0.8**	**0.79**	**0.78**	**0.79**
	Roberta-base	0.72	0.72	0.72	0.72
	Electra-base-discriminator	0.25	0.5	0.33	0.5
	gpt2	0.78	0.77	0.77	0.77

### Error analysis

**Fig. 7:**
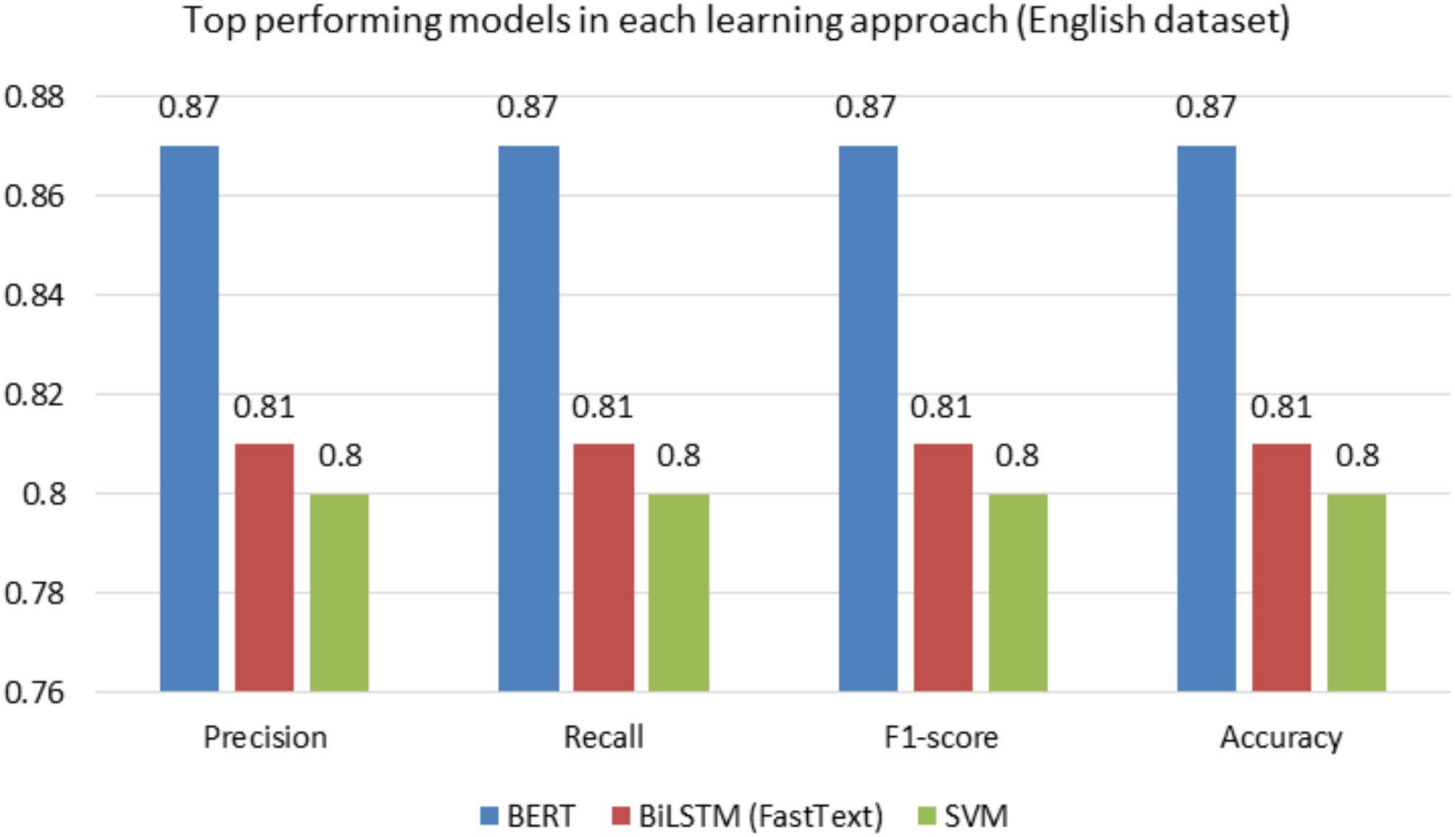
Comparison of the top-performing models in each learning approach of English dataset.

The Fig. 7 showcases the performance of three top-performing models—BERT, BiLSTM (FastText), and SVM—on an English text classification task, evaluated using four metrics: precision, recall, F1-score, and accuracy. Among the models, BERT outperforms the others across all metrics, achieving the highest score of 0.87 consistently. This indicates its exceptional ability to understand and classify English text. BiLSTM (FastText) follows with scores of 0.81 for all metrics, showcasing solid but slightly lower performance compared to BERT. Lastly, SVM performs similarly to BiLSTM, with scores around 0.80, demonstrating its effectiveness but slightly trailing behind. Overall, the figure highlights BERT as the most powerful model for English text, with BiLSTM and SVM as strong alternatives, albeit with slightly lower accuracy and balance.

**Fig. 8:**
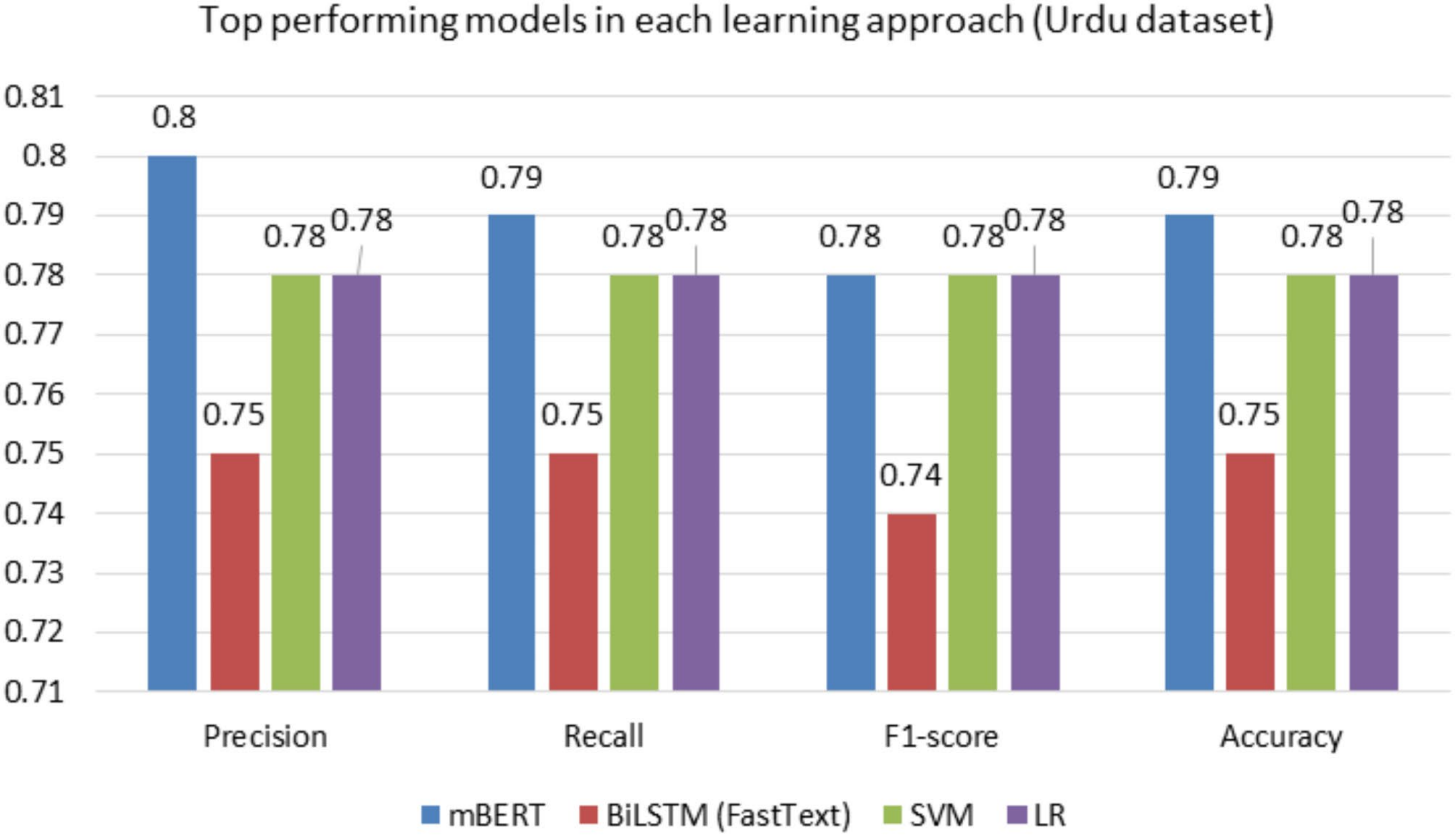
Comparison of the top-performing models in each learning approach of Urdu dataset.

Figure 8 illustrates the performance of four models—mBERT, BiLSTM (FastText), SVM, and LR—on an Urdu text classification task, evaluated across precision, recall, F1-score, and accuracy. mBERT leads in all metrics, with the highest scores of 0.80 for precision and 0.79 for recall, F1-score, and accuracy, showcasing its superior ability to handle Urdu text effectively. SVM and LR follow closely, both achieving consistent scores of 0.78 across all metrics, highlighting their reliability. BiLSTM (FastText), while still competitive, lags slightly behind with scores of 0.75 for recall and accuracy and 0.74 for the F1-score. Overall, mBERT emerges as the top-performing model, demonstrating its strong multilingual capabilities, while SVM and LR provide strong alternatives for Urdu text classification.

Table 9 provides the classification performance of models for two sentiment classes, “hope” and “not hope,” in English and Urdu. For the English dataset, the “not hope” class achieves a precision of 0.88, recall of 0.84, and F1-score of 0.86, with 922 samples. The “hope” class performs slightly better with an F1-score of 0.87 due to a higher recall (0.89) despite slightly lower precision (0.85). The overall accuracy for English is 0.87, indicating strong model performance in distinguishing between the two classes.

**Fig. 9:**
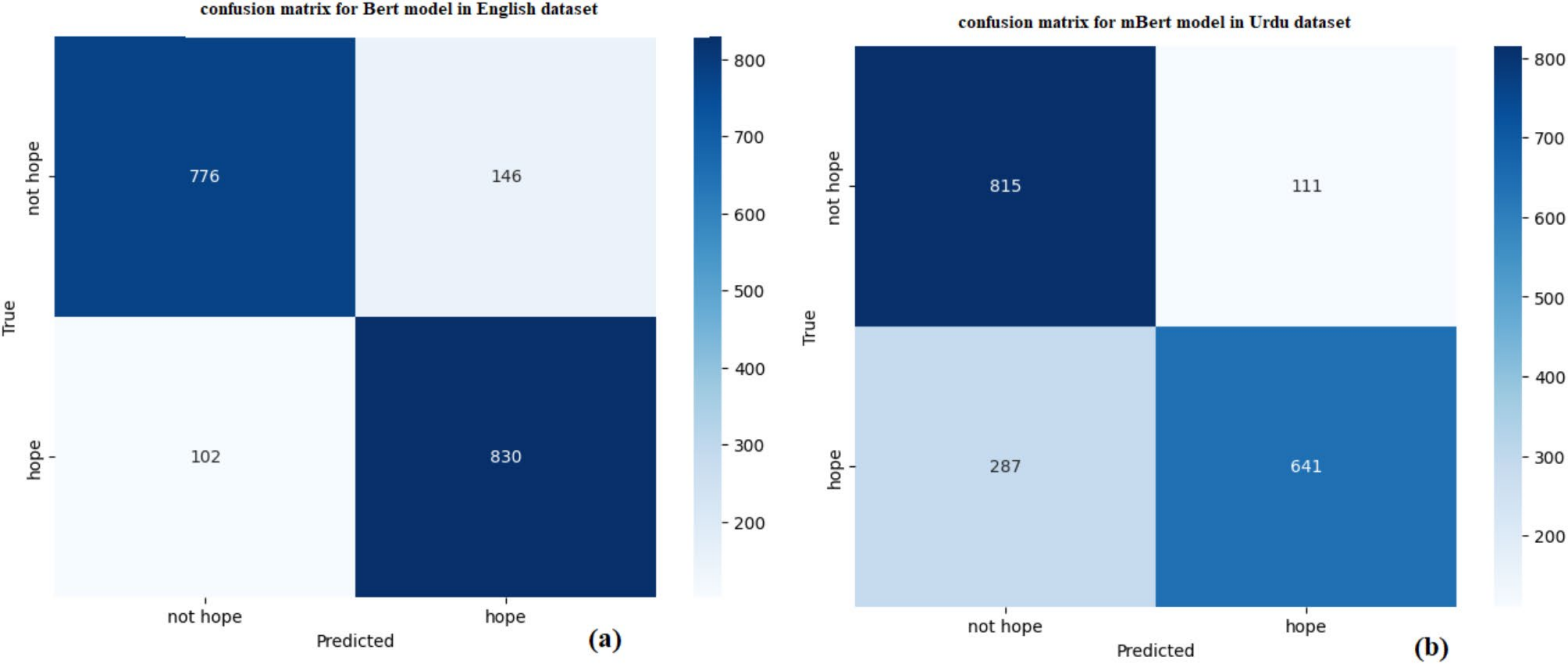
(**a**) Confusion matrix in English and (**b**) shows the confusion matrix of Urdu dataset.

**Fig. 10:**
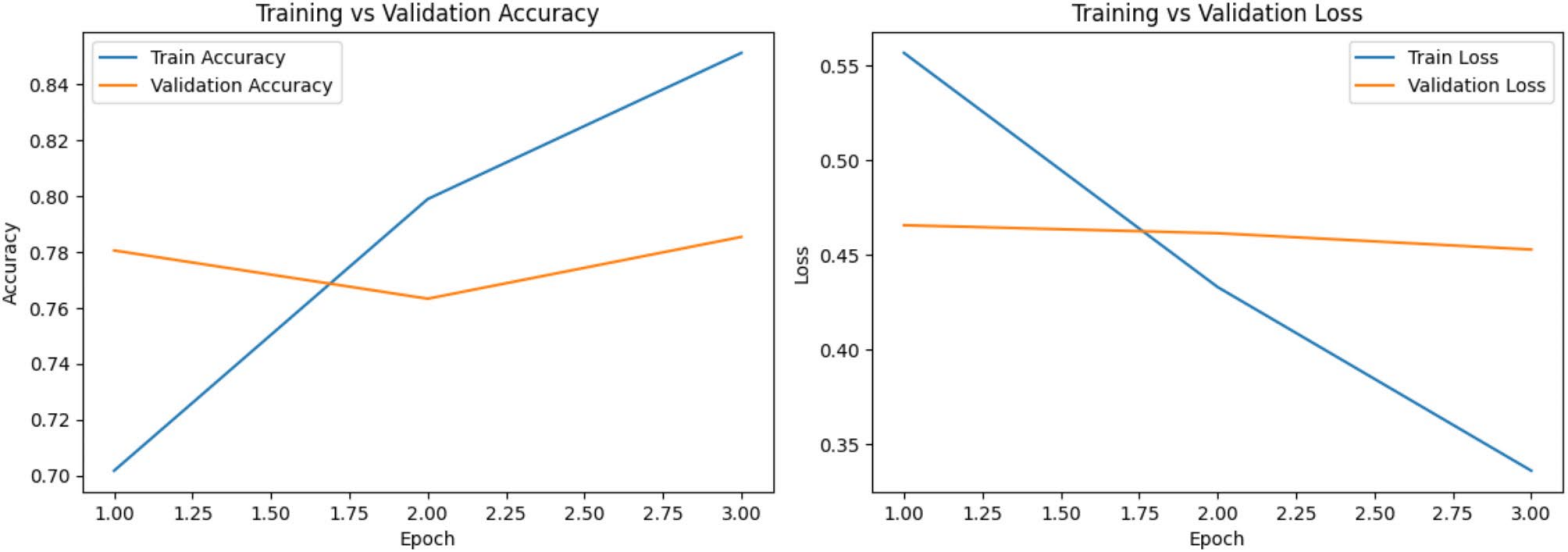
Training and validation performance of different epochs in Urdu.

**Fig. 11:**
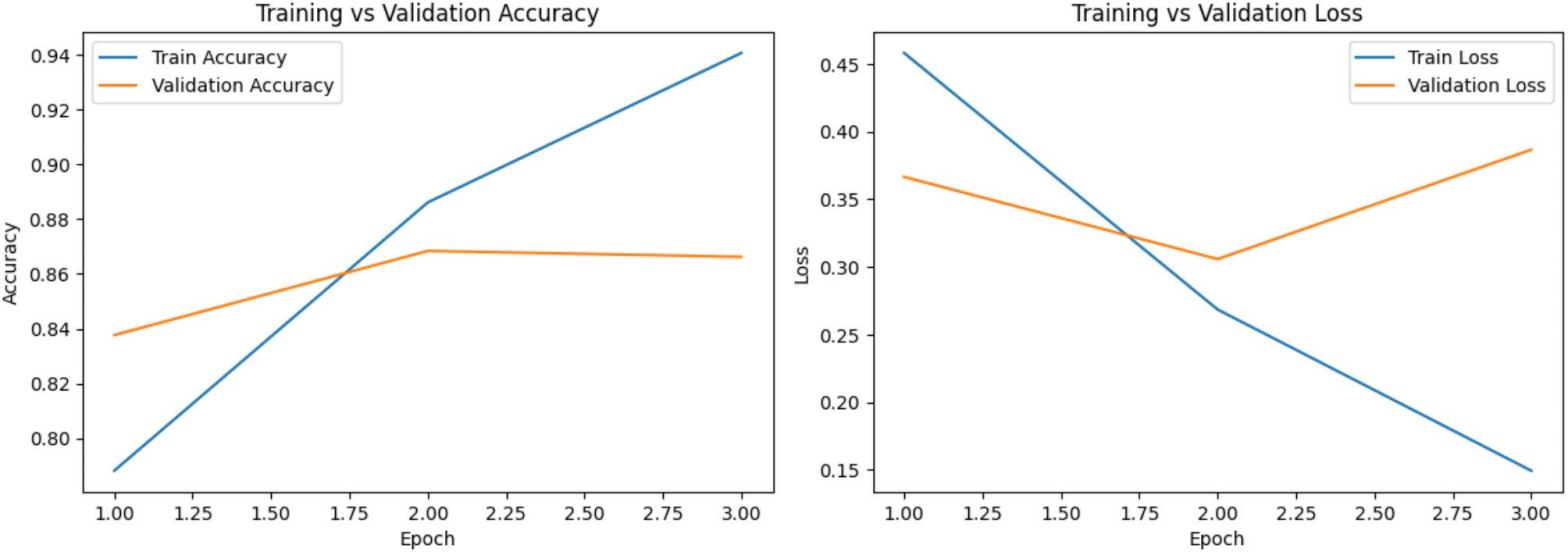
Training and validation performance of different epochs in English.

In the Urdu dataset, the “not hope” class achieves a high recall of 0.88, but precision is lower at 0.74, resulting in an F1-score of 0.80 for 926 samples. For the “hope” class, precision is strong at 0.85, but recall drops to 0.69, yielding an F1-score of 0.76 for 928 samples. The overall accuracy for Urdu is 0.79, which is lower than English, indicating slightly more difficulty in accurately classifying the sentiment in Urdu while Fig. 9a,b shows the confusion matrix and Figs. 10 and 11 shows the comparisons of train vs validation accuracy and train vs. validation loss in both English and Urdu dataset.

Overall, while both languages show strong performance, the model is slightly better at handling English sentiment classification, particularly due to more balanced precision and recall across classes. For Urdu, there is room to improve the recall for the “hope” class and the precision for the “not hope” class.

**Table 9:** Class wise score of BERT model in term of English and Urdu dataset.

Language	Class	Precision	Recall	F1-score	Support	Accuracy
English	Not hope	0.88	0.84	0.86	922	0.87
	Hope	0.85	0.89	0.87	932	
Urdu	Not hope	0.74	0.88	0.8	926	0.79
	Hope	0.85	0.69	0.76	928	

## Limitation of the proposed solution

This study encountered several challenges, especially due to the subjectivity involved in annotating mixed-sentiment tweets in both Urdu and English. A major hurdle in the annotation process was the difficulty of finding native speakers who were fluent in both languages and had the necessary expertise in NLP and machine learning to ensure consistent and accurate labeling. Furthermore, during annotation, tweets with mixed emotional tones presented significant complexities. For example, a tweet like “امید ہے کہ میری مشکلات جلد ختم ہوں گی، مگر کبھی کبھار ایسا لگتا ہے کہ کچھ نہیں بدلے گا” (“I hope my struggles will end soon, but sometimes it feels like nothing will change”) conveyed hope yet contained an underlying sense of despair, making it difficult to assign a definitive label. As Urdu is a low-resource language, there were additional challenges related to the limited availability of annotated data and linguistic tools, which complicated the development of effective machine learning and deep learning models for detecting hope speech. In terms of model performance, despite achieving reasonable success in multilingual hope speech detection (MHSD), the models faced considerable difficulty in classifying tweets with intricate or conflicting emotional tones. For instance, the tweet “میری زندگی میں امید کی کوئی جگہ نہیں ہے، لیکن پھر بھی کچھ کرنے کی کوشش کرتا ہوں” (“There’s no place for hope in my life, but I still try to do something”) illustrates this challenge. These cases highlight a key limitation of the approach: the model’s struggle to accurately capture nuanced sentiments, especially in the context of mixed emotions.

## Conclusion and future work

Social media has become a powerful space for public dialogue, influencing opinions and the emotional landscape of communities. Until now, most research has focused on addressing negativity in the English language, particularly hate speech detection. This study highlights the critical need for multilingual hope speech detection (MHSD) in social media discourse, particularly focusing on the Urdu language, which has been overlooked in existing research. This work addresses a notable gap in current research and underscores the need for more inclusive language processing tools. By exploring a translation-based approach, we aim to tackle the challenges of multilingualism and improve communication across different backgrounds. By creating a multilingual dataset and employing a transfer learning paradigm with fine-tuning, we effectively addressed the challenges associated with identifying hope speech in both English and Urdu. The results indicate that our proposed framework, utilizing pre-trained BERT and translation-based strategies, significantly outperformed baseline models, achieving accuracies of 87% in English and 79% in Urdu. These findings underscore the importance of promoting positive discourse online and demonstrate the potential of hope speech as a means to foster healthier and more constructive interactions within communities. Future work will focus on expanding both the dataset and language coverage by incorporating Large Language Models (LLMs) to enhance the detection of Hope speech across diverse languages and contexts. By increasing the dataset size and adding more languages, we aim to examine how incorporating more samples of hope speech impacts model performance. Additionally, we plan to explore the potential of LLMs such as (GPT 3.5 Turbo) to capture complex linguistic nuances in multilingual discourse.

## Data Availability

The dataset utilized in this study is not publicly available but can be provided upon reasonable request. Interested researchers should contact the corresponding author at Sidorov@cic.ipn.mx, Centro de Investigación en Computación, Instituto Politécnico Nacional (CIC-PN), Mexico City 07738, Mexico. Requests must in-clude a detailed description of the intended use and the requester’s institutional affiliation.
